# Army Correspondence

**Published:** 1862-10

**Authors:** E. Andrews

**Affiliations:** Surgeon of 1st Ill. Light Artillery; Memphis, Tenn.


### ARTICLE XXXII.

MEMPHIS, TENN., *Oct.* 21, 1862.

MESSRS. EDITORS:—Sickness at sometimes, and excess of duties at others, have prevented me from making my usual reports to you of late, concerning army medical matters. The most recent surgical information which I can present is that of the Battle of Corinth.

Orders were there given to amputate no thigh above the middle, without a full council, and then only in desperate emergency. This order was given in consequence of the horrible mortality of high amputations. The result was strikingly, but perhaps fallaciously, brilliant. Of all the thighs amputated below, or at the middle, four-fifths were alive *and doing finely*, on the tenth day, when last heard from. This was among the Union troops. Among the wounded Secesh, who fell into our hands, the same rule was adopted, but the result was exactly reversed. *Four-fifths of similar cases among them died before the tenth day.* This difference in the two classes is due, I believe, to two causes: 1st. The Confederate troops were nearly in a state of starvation, many of them having only roasted green corn in their haversacks. 2d. It is probable that many of the most favorable cases for the operation contrived to crawl away and get carried off on the retreat. There may also be a natural difference in their power of endurance, for it is noticeable through this whole region that the inhabitants have a sallow, thin appearance, which contrasts strongly with the ruddy robustness of our soldiers. In most parts of this region, a ruddy native is a wonder, and a fat one could not be found at all.

Some inquiries into the fate of the compound fracture of the femer above the middle, which by the above rule were excluded from amputation, lead me to doubt the wisdom of the order. They were simply put into the easiest position possible, and kept under expectant treatment only. The result was, that *they all died.* Now, the results of high amputations are frightful, being 80 per cent of deaths; but I submit that even that is better than the death of the whole, as shown under this order. I am inclined to think, from some cases which have fallen under my observation, that gunshot wounds fracturing the femur in the upper fifth, where it consists of cancellar tissue, are less fatal than those of the shaft. At least, one officer whom I treated in such a case with splints made a good recovery.

On the whole, the important question of what shall be done with gunshot compound fractures resolves itself, according to my military experience, as follows:

In the upper half of the femur, dress with extension splints, and, if necessary, make early and free incisions, but do not amputate if it can be avoided.

In the lower half or at the knee-joint amputate or resect.

In the leg, if the knee is perfect, try to save the limb, except in very bad cases.

In the arm and forearm go all lengths to save the limb. Resections are very successful in every part of the superior extremities.

If the shaft of a long bone is shattered by a bullet, and amputation is not performed, free incisions are usually required, either immediately or after the third day. The bullet hole is apt to close, or be blocked with shreds of flesh; the pus is confined, and burrows horribly up and down among the muscles, and forms so large a suppurating cavity that the patient succumbs from exhaustion. A large proportion of the patients, however, die of prostration before suppuration commences.

The health of the army here has been excellent. Among 9000 men, in the month of September, the sickliest of the year, the deaths were only 47. About 300 are in hospitals, and 500 slight cases receive medical attention in quarters. The deaths are only about 1 per cent of all the cases which come under treatment.

Dysenteries have not been severe, as a general rule. I have had about 150 cases in the artillery, since entering the service, and not a single one has died. I lost a few patients of chronic diarrhœa, but none of the acute forms. There has been no cholera or yellow fever in camp, and only a few cases of congestive chills.

There are about 1400 contrabands here, at work upon the fortifications. About 1 or 2 per cent of them have pistol wounds, received in escaping from their masters. Of course, the severer cases do not reach here; hence we see a good many moderate wounds among them. One of them was shot through the lungs and in the hand, and had some fifty dog bites on his legs, and a spot beaten raw on his back, of the size of a dinner plate. I tried to save him, but he died of his injuries, on the third day after he came in. Two days ago, the guerillas shot a negro in sight of the fort on the opposite side of the river, and left his body unburied on the sand. Our soldiers went over and interred it. The barbarities of this sort, which have fallen under my notice, have constituted more than half my surgery, for the last two months. The skirmishes with the enemy in this vicinity, result mostly in buckshot wounds upon our soldiers, which are of no great severity. One case, however, where the shot was made close at hand, sent a projectile into the brain, of which the patient died in about five days.

The foolhardiness of some men is amazing. At Fort Randolph, I saw three officers take a loose torch and explore an old subterranean rebel magazine, where were scores of charged shells lying about, and the torch dropping fire among them. An artillery soldier came to me with a wounded hand. He had been trying to burn out the fuze of an old rebel shell with a red hot poker. The success of his interesting experiment astounded him.

The negroes at work upon the fortifications have about the same proportion of sick as the soldiers, and exactly the same diseases, viz.: intermittents, remittents, diarrhœas, and dysenteries. I think the question of their capacity to tolerate climatic influences may be answered thus: they endure the heat of summer with more comfort than the whites, but are not a particle more exempt from malarious diseases. My observations here confirm those of Dr. Cartwright, of New Orleans, in that respect. In cases of dysentery, I think they succumb to the disease more quickly than the whites.

The Surgeon-General has commenced a pathological cabinet at Washington, and ordered all specimens collected in the army to be forwarded. It is a worthy object, and may be the means of throwing great light on some points of surgery, especially the effects of projectiles upon the long bones.

I trust I shall be with you to resume my lectures in Lind University, this winter. Yours truly, E. ANDREWS.

Surgeon of 1st Ill. Light Artillery.

